# Therapeutic Modulation of Virus-Induced Oxidative Stress via the Nrf2-Dependent Antioxidative Pathway

**DOI:** 10.1155/2018/6208067

**Published:** 2018-10-31

**Authors:** Choongho Lee

**Affiliations:** College of Pharmacy, Dongguk University, Goyang 10326, Republic of Korea

## Abstract

Virus-induced oxidative stress plays a critical role in the viral life cycle as well as the pathogenesis of viral diseases. In response to reactive oxygen species (ROS) generation by a virus, a host cell activates an antioxidative defense system for its own protection. Particularly, a nuclear factor erythroid 2p45-related factor 2 (Nrf2) pathway works in a front-line for cytoprotection and detoxification. Recently, a series of studies suggested that a group of clinically relevant viruses have the capacity for positive and negative regulations of the Nrf2 pathway. This virus-induced modulation of the host antioxidative response turned out to be a crucial determinant for the progression of several viral diseases. In this review, virus-specific examples of positive and negative modulations of the Nrf2 pathway will be summarized first. Then a number of successful genetic and pharmacological manipulations of the Nrf2 pathway for suppression of the viral replication and the pathogenesis-associated oxidative damage will be discussed later. Understanding of the interplay between virus-induced oxidative stress and antioxidative host response will aid in the discovery of potential antiviral supplements for better management of viral diseases.

## 1. Virus-Induced Oxidative Stress

Utilization of oxygen as a final electron acceptor in the mitochondrial energy metabolism by a cell leads to inevitable generation of oxygenated byproducts. Due to their high reactivity, they are often referred to as “reactive oxygen species” (ROS). Typical examples of them include superoxide (O_2_^−^), hydrogen peroxide (H_2_O_2_), hydroxyl radical (OH^−^), and singlet oxygen [1]. In general, most of ROS are considered harmful due to their cell-damaging effects. Therefore, a variety of cellular defensive measures are designed in place to control them. In general, oxidative stress is defined as an imbalance between prooxidant and antioxidant systems. The surplus amount of oxidative stress results in cellular damage due to the oxidation of various essential host macromolecules. A series of so-called “phase II cytoprotective and detoxifying enzymes” are in charge of a mission to serve as a cellular guardian. A different kind of microbial infections was also shown to be a strong inducer of oxidative stress [2–5]. Interestingly, this infection-initiated oxidative stress was demonstrated to play a key role in the activation of innate immunity to fight off pathogenic microbes [6, 7]. Therefore, proper management of cellular oxidative stress is an important cellular task not only for the preservation of its cellular components but also for the maintenance of a germ-free state in the host cell.

Understanding of the mutual interaction between a host and a virus is a prerequisite to developing an effective antiviral strategy to control viral infection. Due to its limited genome size, a virus has evolved a unique talent to take a full advantage of host cellular environments to its favor. This means that all the requirements necessary for the successful completion of a viral life cycle are completely satisfied at the expense of a variety of host metabolic processes. However, these virus-induced changes on a host cellular metabolism have to be in a tight control in order to minimize their potentially detrimental effects on the overall health of the infected host cell. Interestingly, a number of viruses were shown to induce oxidative stress on purpose to facilitate their replication inside the cell [8–14]. This virus-induced oxidative stress and its proper management by a host cell might be a perfect example to understand the harmonious balance between a host and a virus. As previously mentioned, several critical antiviral signaling pathways such as Toll-like receptor and interferon (IFN) pathways are initiated by infection-induced oxidative stress [15, 16]. In addition, defective maintenance of an appropriate redox balance by a host cell has been shown to contribute to the viral pathogenesis, resulting in massive induction of the oxidative stress-induced cell death [17–19]. Basically, the imbalance between ROS production and antioxidant defense system is in a direct link with the disruption of normal cellular physiology [20–22]. A growing number of papers have described this virus-induced oxidative stress as one of the major pathogenic mechanisms for inflammatory response and tissue injury by a viral infection [23, 24]. Therefore, a better understanding of the relationship between virus-induced oxidative stress and antiviral response of a host cell will be a prerequisite for the development of an effective therapeutic strategy to combat various viral diseases.

## 2. Activation of the Nrf2 Pathway as a Defensive Mechanism against Oxidative Stress

**Figure 1:**
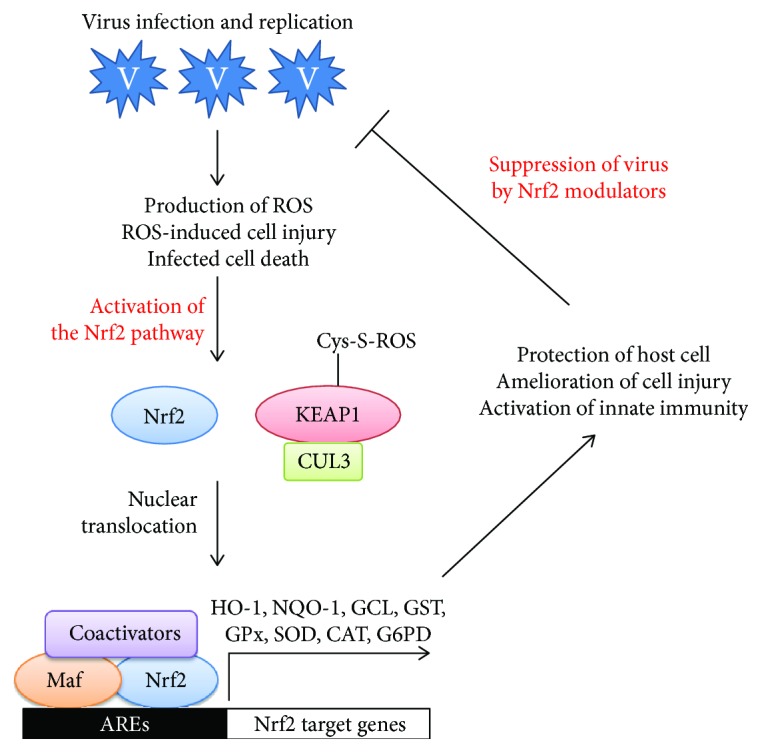
Induction of oxidative stress by a virus infection and genome replication. Subsequent activation of the Nrf2 pathway and amelioration of oxidative stress-induced cellular injury. Abbreviations used within the figure are as follows. Nrf2: nuclear factor erythroid 2-related factor 2; KEAP1: Kelch-like ECH-associated protein 1; CUL3: cullin-3; AREs: antioxidant response elements; HO-1: heme oxygenase-1; NQO-1: NAD(P)H quinone oxidoreductase-1; GCL: glutamate cysteine ligase; GST: glutathione; GPx: glutathione peroxidase; SOD: superoxide dismutase; CAT: catalase; G6PD: glucose 6 phosphate dehydrogenase.

In order to neutralize the deleterious effects of oxidative stress, mammalian cells established a unique antioxidative defense system. This system is designed to be turned off under a normal condition. However, upon encountering oxidative stress, an oxidant-sensitive molecule is activated and transcriptionally stimulate a series of genes responsible for cytoprotection and detoxification. Nuclear factor erythroid 2p45-related factor 2 (Nrf2) is a transcription factor, which has evolved for this purpose. It is one of the best-characterized antioxidative transcription factors with an oxidants/electrophile-sensor function [25]. This basic leucine zipper protein consists of six conserved Nrf2-ECH homology (Neh) domains [26]. Under normal condition, it forms a complex with Kelch-like ECH-associated protein 1 (KEAP1), a well-known negative regulator of Nrf2 [27]. Since KEAP1 serves as an adaptor protein for cullin-3-based E3 ubiquitin ligase, this dimeric Nrf2/KEAP1 complex subjects Nrf2 to constant ubiquitination and proteasomal degradation [28]. In regard to their oxidant-sensing mechanism, redox-sensitive twenty-five cysteine residues of KEAP1 were shown to play a key role in the regulation of the E3 ubiquitin ligase activity [29]. Basically, these cysteine residues are very susceptible to conjugation of a variety of ROS-inducing agents. Once conjugated, the KEAP1-mediated ubiquitination of Nrf2 was severely diminished [30]. This leads to liberation of Nrf2 from the KEAP1-mediated restraint. Once stabilized, Nrf2 is able to get inside the nucleus and form a complex with one of small Maf proteins and other coactivators. Then binding of this trimeric complex to the antioxidant response elements (AREs) in the promoter regions facilities transcription of a series of cytoprotective and detoxifying genes. Typical examples of these Nrf2-target genes include heme oxygenase-1 (HO-1), NAD(P)H quinone oxidoreductase-1 (NQO-1), glutamate cysteine ligase catalytic and regulatory subunits (GCLC and GCLM), glutathione S-transferase (GST), uridine diphosphate glucuronosyltransferase (UDPGT), superoxide dismutase (SOD), catalase (CAT), glucose 6 phosphate dehydrogenase (G6PD), and glutathione peroxidase-1 (GPx) (Figure 1) [31–34].

## 3. Positive and Negative Regulations of the Nrf2 Pathway by Viruses

A virus needs to express a number of nonstructural and structural proteins inside a host cell to support the viral genome replication and assembly of a new virion. Several viral proteins have been shown to be responsible for the production of various ROS [10, 11, 13, 22, 35–40]. This virus-induced oxidative stress plays a central role not only in the successful completion of the viral life cycle but also in the overall viral pathogenesis [20, 35, 41]. However, too much oxidative stress will be a burden on the host cell. Therefore, a virus needs to keep oxidative stress in an optimal level, which should be high enough to support the viral metabolism and should not be too high enough to kill off a host cell. In a way to control the ROS level, a virus has evolved to gain the ability to manipulate the Nrf2 pathway to its favor. Many studies found examples of positive modulation of the Nrf2 pathway by virus-induced oxidative stress [42–48]. However, in some cases, a number of viruses were shown to actively suppress the Nrf2 pathway [20, 43, 49–53]. Here, I would like to introduce evidence of positive and negative modulations of the Nrf2 pathway by various clinically relevant viruses and their implications in the virally induced pathogenesis. Detailed effects of each virus on the ROS level, redox defense system, Nrf2, and its target genes are summarized in Table 1.

### 3.1. Moloney Murine Leukemia Virus ts1

Moloney murine leukemia virus (MoMuLV) ts1 is a mutant retrovirus used for the study of a progressive neurodegeneration induced by a human immunodeficiency virus (HIV) [54, 55]. Accumulation of an incorrectly processed viral envelop glycoprotein, pPr80env, the subsequent onset of ER stress, and the following oxidative stress-induced apoptosis of the infected microglia and astrocytes have been attributed to this virally induced neurodegeneration [56]. However, a selected population of infected astrocytes was able to survive the cytopathic effects of a viral infection [57]. Particularly, upregulation of the antioxidant defense system via activation of the Nrf2 pathway was suggested as a major mechanism for survival of the infected astrocytes [58]. In this report, a significant increase in the levels of Nrf2 and its transcriptional target genes including cell surface cysteine-glutamate antiporter (xCT), glutamate cysteine ligase catalytic and regulatory subunits (GCLC and GCLM), and glutathione peroxidase (GPx) was observed in these cells. In addition, they were shown to harbor the enhanced amounts of redox defense-related proteins such as gamma-glutamyl transpeptidase (*γ*-GT) and catalase [58]. Overall, they were able to maintain much higher levels of intracellular glutathione (GSH) and cysteine relative to those uninfected [58]. Based on these observations, authors concluded that successful immobilization of the thiol redox defense system via a positive modulation of the Nrf2 pathway contributed to the survival of the infected astrocytes despite the cytotoxic effects of MoMuLV ts1 [58].

### 3.2. Human Immunodeficiency Virus Type 1

Human immunodeficiency virus type 1 (HIV-1) plays an etiological role in the development of acquired immunodeficiency syndrome (AIDS). Besides its immune-compromising effects, HIV-1 infection is also linked with the development of neurocognitive disorders [59, 60]. Particularly, a viral gp120 protein was suggested to play a causative role in the HIV-1-associated neurodegeneration through induction of oxidative stress [8]. In response to this virus-induced oxidative stress, the Nrf2 pathway was found to be activated, leading to the expression of a series of the Nrf2-dependent antioxidant response genes including HO-1 and NQO-1 [8]. Pretreatment with both antioxidants and a calcium chelator antagonized this gp120-induced Nrf2 activation, further implicating the active involvement of oxidative stress and calcium signaling in mounting an antioxidative defense measure [8]. Based on these observations, the Nrf2 pathway was suggested to play a protective role in promoting survival of the HIV-infected astrocytes [8].

In addition to the gp120, one of three regulatory viral genes, a Tat protein also has been shown to be responsible for induction of oxidative stress upon HIV-1 infection [61–64]. In regard to one of the potential mechanisms for the Tat-induced oxidative stress, a direct involvement of the N-methyl-D-aspartate (NMDA) receptor and spermine oxidase (SMO) was proposed [65]. In this report, stimulation of the NMDA receptor by Tat and the subsequently accelerated catalysis of spermine into spermidine by SMO were suggested as the main mechanism for an increased production of H_2_O_2_ [65]. This, in turn, stabilized Nrf2 and transactivated the Nrf2 target genes such as NQO-1, CAT, SOD1, and HO-1 [65]. These series of cytoprotective protein and enzymes ultimately prevented the HIV-1 Tat-induced cell death [65]. Based on these findings, the Nrf2 pathway was proposed as an important determinant for protection against HIV-1-induced neurodegeneration [65].

The pulmonary complication is one of the leading causes of death by HIV patients [66]. In particular, HIV-1 transgene expression was shown to significantly impair alveolar macrophage phagocytic capacity in the HIV-1 transgenic rats [67]. In relation to this observation, HIV-1 gp120 and Tat were identified as causative agents for oxidative stress and glutathione depletion by HIV-1 infection [38]. However, contrary to previous reports, Nrf2 expression was decreased in alveolar epithelial cells from HIV-1 transgenic rats compared with their wild-type counterparts [68]. This diminished expression of Nrf2 further increased epithelial barrier permeability and decreased transepithelial electrical resistance in HIV-1 transgenic rats [51]. Suppression of the Nrf2 pathway was also detected in human monocyte-derived macrophages either infected with HIV-1 or exposed to HIV-related proteins [53]. In line with this observation, accelerated aging by HIV-1 infection was noticed in the HIV-1 transgenic rats [50]. In these HIV-1 transgenic rats, a significant reduction in the protein levels of Nrf2 and HO-1 was also confirmed [50]. This further implicates the redox imbalance induced by expression of HIV-1 transgenes as a causative for the promotion of senescence in the transgenic rats [50]. Based on these findings, the use of Nrf2 activators was suggested as a promising approach to enhance lung innate immunity in HIV patients [53].

Transcriptional regulation of HIV-1 genes is controlled by a distinct viral enhancer and promoter called “long terminal repeats” (LTRs). A variety of host and viral transcriptional factors bind this region in a concerted manner to ensure a fine-tuning of viral gene expression. Particularly, Tat was shown to play an essential role in a positive regulation of the LTR-dependent transcription of viral genes [61]. Together with gp120, Tat is one of two main players responsible for the production of increased levels of ROS and subsequent activation of the Nrf2 pathway in the HIV-1-infected cells. Activation of the Nrf2 pathway by Tat was further manifested by enhanced transcription of the downstream Nrf2 target genes such as NQO1, HO-1, and aldo-ketoreductase 1C1 (AKR1C1) [63]. Interestingly, the Nrf2 pathway turned out to play a critical role in the inhibition of the LTR-dependent transcription of viral genes [63]. These findings suggested a pharmacological modulation of the Nrf2 pathway as a plausible antiviral strategy for the abrogation of the LTR-dependent transcription by HIV [63].

### 3.3. Hepatitis C Virus (HCV)

HCV infection is responsible for the development of chronic hepatitis [69]. Several HCV proteins, including core, NS3, and NS5A, have been ascribed to induction of oxidative stress in human hepatoma cells [36, 37, 70, 71]. Hepatocellular damage from HCV has been linked to HCV-induced oxidative stress. [35]. On the other hand, production of ROS by HCV infection was shown to induce phosphorylation and nuclear translocation of Nrf2, thereby transactivating its target genes such as NQO1, HO-1, and *γ*GCSH [48]. Particularly, cellular kinases including JNK, ERK1/2, p38 mitogen-activated protein kinases (MAPKs), phosphatidylinositol 3-kinase/Akt (PI3K-Akt), protein kinase C (PKC), and casein kinase 2 have been implicated in phosphorylation and activation of Nrf2 [72–75]. Based on these findings, the activation of the Nrf2 pathway was proposed to be one of the potential mechanisms for the survival of HCV-infected cells [48].

Chronic hepatitis C was shown to be in a frequent association with steatosis, accumulation of lipid droplet [76]. In order to study the HCV-induced steatosis, Sugiyama et al. successfully established a persistently infected hepatoma cell line and maintained it for more than a year [77]. As expected, a remarkable accumulation of lipid droplets was detected in this cell line [77]. Integrated analysis of metabolomics and expression arrays revealed a constitutive upregulation of the Nrf2 pathway-associated genes including NQO1, GCLC, Maf, glucose 6 phosphate dehydrogenase (G6PD), methylenetetrahydrofolate dehydrogenase 2 (MTHFD2), and asparagine synthetase (ASNS) [77]. In particular, Nrf2 phosphorylation was also found to be increased in the nuclear extract of these cells [77]. Knockdown of Nrf2 significantly suppressed steatosis and HCV infection [77]. These findings imply a negative modulation of the Nrf2 pathway as a promising strategy to dampen the HCV-induced steatosis.

Nrf2-dependent metabolic reprogramming and its positive implication in the HCV disease progression were examined by Saito et al. [78]. In this study, a phosphomimetic version of p62, which is a negative modulator of KEAP1, was designed and expressed [78]. Interestingly, this phosphomimetic p62 was found to activate the Nrf2 pathway [78]. This, in turn, facilitated the malignant progression of hepatocellular carcinoma (HCC) by HCV [78]. Downstream Nrf2 target genes such as phosphogluconate dehydrogenase (PGD), GCLC, NQO1, and UDP-glucose dehydrogenase (UGDH) were all found to be upregulated at the transcriptional level by the phosphomimetic p62 [78]. Consequently, this p62-dependent Nrf2 activation gave rise to a robust GSH production, resulting in tolerance to anticancer drugs and enhanced proliferative capacity in infected hepatoma cells [78]. Based on these findings, a specific inhibitor for KEAP1 and phospho-p62 interaction, K67, was identified by a high-throughput screening [78]. This Nrf2 inhibitor was able to suppress tumor growth and tolerance to anticancer agents, further confirming the molecular targeting of p62 as a potential chemotherapeutic strategy to combat HCC by HCV [78].

As previously noted, HCV associated with oxidative stress leads to the activation of the Nrf2 pathway [72–75]. In contrast to this, Carvajal-Yepes et al. discovered a unique mechanism for active downregulation of the Nrf2 pathway by an HCV infection [49]. According to their study, the viral proteins such as core and NS3 were able to induce mislocalization of small Maf, which is a partner transcription factor for Nrf2, resulting in inhibition of Nrf2-dependent expression of target genes such as NQO1, GCLC, and GPx [49]. Overall proteasomal activity was also downregulated in the infected cells [49]. Therefore, depending on the cellular contexts and levels of oxidative stress, HCV infection seems to exert a differential influence on the Nrf2 pathway [49].

### 3.4. Influenza Virus

Influenza viruses have been demonstrated to be a causative agent for oxidative stress and respiratory inflammation by a number of studies [41, 79–82]. In line with these observations, an influenza virus was shown to induce apoptosis and cytotoxicity in alveolar epithelial cells, as manifested by an increased expression of caspase 1, caspase 3, and a proinflammatory cytokine, IL-8 [44]. Therefore, attenuation of oxidative stress and inflammation by a pharmacological measure may be beneficial for lessening an influenza virus-induced lung injury and exacerbation of existing respiratory diseases. Interestingly, this influenza virus-induced oxidative stress, in turn, was able to activate the Nrf2 pathway through the facilitation of the nuclear translocation of Nrf2 and subsequent expression of the Nrf2 target genes like HO-1 in the human alveolar epithelial cells [44]. Consequently, induction of the Nrf2 downstream genes was able to protect the infected cells against virus-induced cellular injury [44]. Similar lung injury induced by lipopolysaccharide (LPS) was also shown to be alleviated by activation of the Nrf2 pathway [83]. However, in contrast to this observation, a proteomic analysis performed by Simon et al. found a negative impact of the influenza virus infection on the Nrf2 pathway [84]. In this report, virus-infected human bronchial adenocarcinoma cells were found to have a lower amount of a phosphorylated form of Nrf2 in their nuclei [84]. Similar to HCV infection, this cellular context-dependent differential modulation of virus-induced antioxidative responses seems to be another recurring theme in the case of the influenza virus.

### 3.5. Respiratory Syncytial Virus

Respiratory syncytial virus (RSV) is responsible for viral upper and lower respiratory tract infections in infants and young children [85]. RSV infection is associated with the development of severe lower respiratory illness associated with bronchiolitis and respiratory failure [85]. RSV infection of airway epithelial cells was shown to induce ROS production, which is involved in transcription factor activation and chemokine gene expression for inflammation and innate immune defense [86, 87]. In line with these results, RSV infection was reported to induce a significant increase in lipid peroxidation products as well as a significant decrease in the GSH/GSSG ratio in human alveolar type II-like epithelial cells and small airway epithelial cells [20]. However, in spite of virus-induced oxidative stress, RSV infection was able to abrogate activation of the Nrf2 pathway, resulting in a reduction in the expression levels of Nrf2 target genes including HO-1, SOD1, SOD3, GST, CAT, and GPx [20]. Consequently, the virus-induced cellular oxidative damage was further accelerated [20, 88]. Although another group of researchers reported activation of the Nrf2 pathway by RSV infection, as verified by the nuclear translocation of Nrf2 and the increased expression of Nrf2 target genes such as GCLC, UGT1, NQO1, HO-1, GST, and GPx in the normal mice, this Nrf2 activation was very transient and disappeared only one day after RSV infection [19]. In addition, lack of Nrf2-dependent antioxidant expression in mice genetically deficient in Nrf2 was also shown to exacerbate lung inflammation and injury [19]. In line with this, analysis of bronchoalveolar lavage proteins retrieved from RSV-infected mice revealed a global reduction in expression of antioxidant enzymes including SOD1, SOD3, CAT, GST, and GPx through inactivation of the Nrf2 pathway [20, 89]. Collectively, RSV seems to be gifted with a special power for active downregulation of the Nrf2 pathway to facilitate its pathogenesis.

In regard to potential mechanisms for inactivation of the Nrf2 pathway by RSV, dysregulation of posttranslational modification of Nrf2 was suggested [90]. Basically, RSV-induced deacetylation, SUMOylation, and the following proteasomal degradation of Nrf2 were shown to be responsible for downregulation of transcription of Nrf2-dependent genes such as NQO1, CAT, and SOD1 [90]. In particular, a SUMO-specific E3 ubiquitin ligase, RING finger protein 4 (RNF4), was shown to play a central role in the process of RSV-induced Nrf2 degradation [43]. In support of this mechanism, treatment of the histone deacetylase (HDAC) inhibitor, trichostatin A (TSA), significantly facilitated acetylation and degradation of Nrf2 [90]. In addition, RSV infection gave rise to a significant reduction in binding of the transacetylase, CBP, to the ARE site of the SOD1 gene promoter [90]. Based on these findings, a pharmacological recuperation of the Nrf2 pathway by an Nrf2 activator could be employed to produce ameliorating effects on the RSV-induced pathogenesis.

### 3.6. Hepatitis B Virus (HBV)

HBV is regarded as one of the major etiological factors in the development of HCC [91]. Accumulating evidence has suggested a constitutive activation of the Nrf2 pathway in various human cancers [92–96]. In many tumors, increased expression of Nrf2 target genes was considered beneficial for tumor cells' escape from chemotherapy-induced cytotoxicity through upregulation of antioxidative response [97, 98]. Chronic inflammation and concomitant cellular stress due to permanent overproduction of ROS-inducing viral proteins have been associated with the development of HCC by HBV [15, 16]. HBV infection was reported to induce a strong activation of the Nrf2 pathway [99]. More specifically, HBV protein X (HBx) and large HBV surface protein (LHB) were shown to be responsible for activation of the Nrf2 target genes such as NQO1, GPx, and GCL [99]. This HBV-dependent induction of the Nrf2-regulated genes seems to protect infected cells from oxidative damage [99]. In regard to a potential mechanism for HBx-induced activation of the Nrf2 pathway, ATM kinase, which is a well-known DNA damage sensor, was implicated in this process [100]. In this report, HBx-induced ROS generation increased a phosphorylated form of ATM, resulting in facilitating the Nrf2-dependent transcription of HO-1, NQO1, and G6PD genes [46, 100]. In this report, HBx was able to augment the interaction between KEAP1 and p62. Since p62 is a negative regulator of KEAP1, increased association of KEAP1 with p62 by HBx liberates Nrf2 from the KEAP1-Nrf2 complex, leading to the activation of the Nrf2 pathway. Consequently, transcription of Nrf2 target genes including G6PD, NQO1, GST, and Cyp2a5 was increased [46]. Upregulation of another Nrf2 target gene, the insulin receptor, by HBV infection was also described [101]. However, in contrast to a robust induction of Nrf2 by HBV infection, infection by HBV genotype G was shown to inhibit activation of the Nrf2 pathway due to intracellular accumulation of subviral HBsAg particles [52]. Levels of the Nrf2 target genes such as NQO1, AP1, and GPx were also significantly decreased in these HBV/G replicating cells [52]. In addition to the influenza virus and RSV, differential modulation of the Nrf2 pathway based on cellular contexts also seems to be applicable in the case of the HBV infection.

### 3.7. Herpes Virus

ROS production and its associated oxidative tissue damage have been shown to play a causative role in herpes simplex virus- (HSV-) 1-induced neuropathology [102–104]. Subsequent antioxidant gene induction was also observed during experimental herpes encephalitis [105]. Particularly, upregulation of the Nrf2 target genes such as HO-1 and GPx was confirmed in the herpes disease model [105]. In this report, astrocytes were shown to mediate antioxidative stress response upon HSV-1 infection [105]. Another type of herpes virus, human cytomegalovirus (HCMV), also demonstrated activation of the Nrf2 pathway for neutralization of the cytotoxic effects of ROS [106]. In this report, HCMV-infected cells have increased levels of Nrf2-dependent antioxidant and detoxifying enzymes such as SOD, GPx, GCLC, HO-1, and NQO1 [106]. This led to an increase in the glutathione levels in the virus-infected cells [106]. Lee et al. also reported a similar result, which described the protection of host cells from oxidative stress via upregulation of Nrf2 expression by HCMV infection [42]. In this study, expression of the Nrf2 target genes such as HO-1 and GCLC was induced by virus immediate early (IE) proteins irrespective of ROS [42]. This suggests existence of a ROS-independent mechanism for activation of the Nrf2 pathway in the case of HCMV infection. Specifically, CK2 kinase was shown to be involved in this HCMV-mediated activation of Nrf2 [42]. Another type of herpes virus, a Kaposi's sarcoma-associated herpesvirus (KSHV), plays an etiological role in the development of Kaposi's sarcoma and primary effusion B-cell lymphoma [107, 108]. KSHV has been implicated in the Nrf2 induction upon infection of endothelial cells [45]. In this report, de novo KSHV infection of human dermal microvascular endothelial cells activated the Nrf2 pathway through the ROS-mediated dissociation of KEAP1 from the Nrf2-KEAP1 complex and subsequent Nrf2 phosphorylation and nuclear translocation [45]. This led to an increased expression of the Nrf2 target genes such as NQO1 and HO-1 [45]. In particular, activated Nrf2 was found to be colocalized with the KSHV genome as well as with the latency protein LANA-1, further suggesting a potential role of Nrf2 in the direct regulation of transcription and replication of KSHV genomes [45].

### 3.8. Dengue Virus

Dengue virus (DENV) is an arthropod-borne tropical virus responsible for the development of dengue fever and related diseases [109, 110]. Study of DENV infection suggests the presence of an important interplay between the generation of oxidative stress and the immunopathology of DENV disease. Preferential activation of the Nrf2 pathway by a DENV infection in primary human monocyte-derived dendritic cells was reported by using a genome-wide transcriptome analysis [39]. In this report, the cellular oxidative stress response is required for the DENV-induced innate immune responses [39]. In particular, accumulation of intracellular NOX-derived ROS in infected cells was required for potentiation of the immune response [39]. The Nrf2 target genes, which are stimulated by a DENV infection, include HO-1, NQO1, SOD2, GCLM, and GCLC [39]. Another example of the activation of the Nrf2 pathway by DENV in mononuclear phagocytes was also described [47]. In this study, DENV NS2B3 protein was shown to be involved in ER stress induction and activation of the Nrf2 pathway. The NS2B3-induced activation of the Nrf2 pathway resulted in upregulation of c-type lectin domain family 5, member A (CLEC5A) and ultimately production of tumor necrosis factor- (TNF-) *α* [47].

### 3.9. Marburg Virus

Marburg virus (MARV) is a causative agent for lethal hemorrhagic fever in humans [111]. Two separate research groups demonstrated activation of the Nrf2 pathway by MARV infection through inhibitory effects of the viral protein VP24 on KEAP1 [112, 113]. In their studies, the VP24 binding site was found to be located within the Kelch domain of KEAP1, which happened to overlap with the Nrf2-binding site [112, 113]. Therefore, expression of VP24 induced Nrf2 activation and transcription of the Nrf2-dependent genes such as HO-1, NQO1, and GCLM [112, 113]. Interestingly, VP24 dimerization was shown to play a role in the regulation of VP24-KEAP1 interaction since the loss of VP24 dimerization resulted in increased KEAP1 binding and VP24-dependent ARE promoter activity [114]. Therefore, pharmacological inhibition of the Nrf2 pathway may be useful for dampening the MARV-associated pathogenesis.

### 3.10. Spring Viremia of Carp Virus (SVCV)

Spring viremia of carp virus (SVCV) is the etiological agent of spring viremia of carp [115]. SVCV infection was able to upregulate the cellular total antioxidant capacity and Nrf2 expression, leading to an increase in the expression of the Nrf2 target genes such as HO-1 and SOD1 [116, 117]. Elevated production of ROS upon SVCV infection seems to be responsible for activation of the Nrf2 pathway [116, 117].

### 3.11. West Nile Virus

West Nile virus (WNV) infection plays an etiological role in the development of neuroinvasive disease such as a mosquito-borne encephalitis [118–120]. WNV infection was also shown to activate the Nrf2 pathway, as evidenced by a significant increase in antioxidant gene expressions such as GCLC, SOD, and GPx [121]. In this report, increased GSH levels via activation of the Nrf2 pathway inhibited arsenite-induced stress granule formation in WNV-infected BHK cells. Based on these observations, authors suggest that WNV-induced activation of the Nrf2 pathway protects infected cells against mitochondrial damage induced by arsenite-induced ROS [121].

### 3.12. Red-Spotted Grouper Nervous Necrosis Virus

Red-spotted grouper nervous necrosis virus (RGNNV), a pathogenic fish virus, induced oxidative stress, apoptosis, and postapoptotic necrosis in a grouper liver cell line [122]. RGNNV infection was shown to be capable of ROS production and subsequent upregulation of antioxidant enzymes such as Cu/Zn SOD and catalase in GF-1 cells [122].

## 4. Therapeutic Modulation of a Viral Pathogenesis via an Nrf2-Dependent Antioxidative Pathway

Some viruses induce oxidative stress on purpose for a successful completion of a virus life cycle. However, uncontrolled virus-induced oxidative stress exacerbates the condition of the infected cells. Therefore, upregulation of the cytoprotective and detoxifying protein seems to be beneficial not only for disruption of the ROS-dependent steps of the viral life cycle but also for the amelioration of the exacerbated conditions of the infected host cells. In this regard, numerous pharmacological agents were shown to activate the Nrf2 pathway and lessen the burden of virus-induced oxidative stress [10, 11, 14, 22, 35–38, 40, 123]. In addition, a number of Nrf2 overexpression and knockdown studies also demonstrated the direct involvement of the Nrf2 pathway in the pathogenesis of some viruses [124–127]. Here, I would like to introduce studies describing the effects of Nrf2 modulators and genetic manipulation of Nrf2 on virus replication and virally induced pathogenesis. Detailed effects of pharmacological and genetic alterations of the Nrf2 pathway on Nrf2 protein, Nrf2 target genes, ROS level, viral pathogenesis, and virus replication are summarized in Table 2.

### 4.1. MoMuLV ts1

*α*-Luminol (monosodium 5-amino-2-3-dihydro-1-4-phthalazine dione) is an anti-inflammatory drug extensively studied by Russian scientists [128]. Jiang et al. demonstrated that treatment with *α*-luminol was able to suppress oxidative stress induced by MoMuLV ts1 infection [40]. In addition, *α*-luminol abrogated upregulation of Nrf2 in MoMuLV ts1-infected astrocytes, resulting in restoration of increased GPx and cell surface cysteine-glutamate antiporter (xCT) to a normal level [40]. This led to the reduction of H_2_O_2_ and recuperation of cellular DNA synthesis and the GSH level [40]. In in vivo animal study, *α*-luminol was able to suppress the neurodegeneration in MoMuLV ts1-infected mice [40]. Particularly, early *α*-luminol treatment even blocked ts1 replication and expression of the gPr80env protein in the CNS of MoMuLV ts1-infected mice [40]. Based on these observations, they concluded that *α*-luminol suppresses virus replication and virus-induced cytopathology in the CNS by reducing oxidant stress [40]. In line with these evidence, Scofield et al. also reported a positive role of *α*-luminol in the preservation of epithelial cell cytoarchitecture and promotion of thymocyte survival in MoMuLV ts1-infected mice [129]. In particular, gPr80env accumulation was completely prevented in mice treated with *α*-luminol [129]. Similar protective effects of *α*-luminol were also observed in the intestines of MoMuLV ts1-infected mice [130]. Treatment of *α*-luminol was able to preserve crypt-villus epithelial organization and allows survival of intestinal T cells in the MoMuLV ts1-infected mice [130]. Interestingly, intestinal epithelial cells were found to contain higher levels of the Nrf2 protein after *α*-luminol treatment [130]. Suppression of ROS by treatment of *α*-luminol seems to be beneficial in retarding the pathogenesis of MoMuLV ts1 infection through Nrf2-dependent neutralization of virus-induced oxidative stress [130]. On the other hand, Kuang et al. also demonstrated a similar neuroprotective effect of minocycline through direct radical scavenging and upregulation of Nrf2-mediated antioxidant defenses [131]. In this report, decreased Nrf2 levels by MoMuLV ts1 were enhanced by treatment of minocycline [131]. However, in spite of treatment of minocycline, a viral titer remained unaltered, suggesting that a virus replication is not the primary target of minocycline [131].

### 4.2. HIV

Epigallocatechin-3-O-gallate (EGCG), the predominant catechin from tea, is known to exert a variety of biological activities [132–135]. In particular, antiviral activities of EGCG were shown in the context of HIV-1 infection [136, 137]. In regard to possible antiviral mechanisms by EGCG, Zhang et al. reported modulation of Tat-induced LTR transactivation by EGCG [64]. In this paper, EGCG was able to induce a significant improvement on the cellular alterations associated with Tat-induced oxidative stress. These beneficial effects of EGCG seem to be mediated by increasing nuclear levels of Nrf2 and decreasing levels of NF-*κ*B [64]. Based on these results, the Nrf2 signaling pathway was suggested as the primary target for prevention of Tat-induced HIV-1 transactivation. A similar antagonizing effect of another natural compound, called tanshinone II A, on Tat-induced HIV-1 transactivation was also reported [62]. Tanshinone II A is a lipid-soluble monomer derivative of phenanthrenequinone extracted from the root of *Salvia miltiorrhiza* (Danshen) [138, 139]. In this study, tanshinone II A was shown to reverse Tat-induced ROS production and downregulation of GSH levels through upregulation of Nrf2 expression [62]. In particular, this inhibition of Tat-induced HIV-1 LTR transactivation by tanshinone II A was dependent on the AMP-activated protein kinase- (AMPK-) nicotinamide phosphoribosyltransferase (Nampt) pathway [62]. Based on this observation, the authors proposed the AMPK/Nampt/SIRT1 pathway as a promising anti-HIV target [62].

As previously noted, HIV-1 transgene expression in rats significantly dampens alveolar macrophage phagocytic capacity [67]. In particular, HIV-1-related viral proteins such as gp120 and Tat seem to be directly involved in this oxidative stress-cytotoxicity through glutathione depletion in target cells [38]. Fan et al. demonstrated downregulation of Nrf2 expression via RNA interference inhibited Nrf2-dependent antioxidant gene expressions such as GSSG, GCLC, GST, glutathione reductase (GSR), and NQO1. These changes induced by lack of Nrf2 were eventually translated into decreased intracellular glutathione levels, increased epithelial barrier permeability, and decreased transepithelial electrical resistance (TER) in alveolar epithelial cells of HIV transgenic rats [51]. In particular, alteration of tight junction protein expression and localization was worsened by the interference of Nrf2 expression [51]. In contrast, Nrf2 overexpression improved epithelial barrier function as well as tight junction expression and localization in alveolar epithelial cells of HIV transgenic rats [51]. These data suggest the importance of the integrity of the Nrf2 pathway in protecting cells against oxidative stress induced by HIV-1 infection.

Sulforaphane (SFN) is an isothiocyanate abundant in cruciferous vegetables. It is famous for its cytoprotective effects shown by numerous in vivo and in vitro studies [140, 141]. SFN has been found to be a powerful activator of the Nrf2 pathway [142–144]. SFN increased Nrf2-regulated cellular antioxidant response such as induction of NQO1 and glutathione and protected alveolar epithelial cells against HIV-1-induced barrier dysfunction [51]. SFN was also shown to inhibit HIV infection of macrophage [145]. In this study, SFN suppressed HIV infection through blockage of HIV envelope-mediated viral entry [145]. In addition, SFN was able to restore the decreased phagocytic function of the HIV-infected alveolar macrophages by stimulating Nrf2-dependent antioxidative functions such as expression of NQO1 and GCLC [53]. Cross et al. also showed another Nrf2 activator, dimethyl fumarate (DMF), was able to suppress HIV replication and macrophage-mediated neurotoxicity [146]. In this report, DMF treatment was able to upregulate transcription of Nrf2 target genes such as HO-1, GPx, and NQO1. It was also able to inhibit HIV replication and HIV-mediated neurotoxicity in human monocyte-derived macrophages [146]. Particularly, induction of HO-1 by DMF was demonstrated to reduce neurotoxin production from human monocyte-derived macrophages [146].

### 4.3. HCV

HO-1 is one of the best-characterized Nrf2 target genes, readily inducible in response to a variety of oxidative stress and cytotoxic insult. HO-1 catalyzes the oxidation of heme to biliverdin (BV), carbon monoxide, and iron [147]. Zhu et al. demonstrated that a genetic or pharmacologic induction of HO-1 led to inhibition of HCV replication through blockage of a viral NS3/4A protease [148]. BV, an end product of heme oxidation by HO-1, was also shown to possess an anti-HCV activity [149]. Since free BV is rapidly reduced to bilirubin by the enzyme biliverdin reductase (BVR) in the hepatocyte, the antiviral activity of BV was significantly enhanced by BVR knockdown [148]. In addition to this mechanism, BV was also shown to interfere with HCV replication by activation of antiviral IFN response including increased expression of oligoadenylate synthetase (OAS), protein kinase R (PKR), interferon- (IFN-) *α*, and heme-regulated eIF2alpha kinase (HRI) [149]. Lucidone, a natural compound, isolated from the fruits of *Lindera erythrocarpa Makino*, was also found to suppress HCV replication by Nrf2-mediated HO-1 induction [150]. In this report, lucidone augmented antiviral IFN response by upregulation of OAS1, 2, and 3 and PKR. It suppressed HCV NS3/4A protease activity through biliverdin production as well [150]. Andrographolide, the most abundant diterpene lactone in the leaves and stems of *A. paniculata* [151, 152], was demonstrated to work against HCV replication through upregulation of HO-1 via the Nrf2 pathway [153]. In this study, andrographolide enhanced the production of biliverdin, resulting in activation of antiviral IFN response and suppression of HCV NS3/4A protease activity [153]. Particularly, p38 MAPK was shown to be involved in this Nrf2-mediated HO-1 increase by andrographolide [153]. In an attempt to stimulate HO-1 activity, cholesterol-lowering drugs, statins, were used to induce expression of the HO-1 in a variety of cells [154–156]. Among them, fluvastatin was shown to inhibit HCV replication by induction of HO-1 [157]. On the other hand, Yu et al. showed suppression of HCV replication by SFN through upregulation of HO-1 expression. In this study, SFN was able to stimulate BV production to activate antiviral IFN response and suppress HCV protease activity [158]. In regard to a more detailed mechanism, SFN was found to stimulate PI3K phosphorylation, which contributed to Nrf2/HO-1-mediated inhibition of HCV replication [158]. Celastrol, a quinone methide triterpene isolated from *Tripterygium wilfordii* [159–161], was also shown to inhibit HCV replication by upregulating HO-1 via the Nrf2 pathway in human hepatoma cells [162].

Although previous data all pointed out inhibitory effects of Nrf2-activating pharmacological agents on HCV replication through different mechanisms, following two studies demonstrated differential effects of the Nrf2 pathway on HCV replication. Sugiyama et al. showed knockdown of Nrf2 neutralized the increasing effect of chronic HCV infection on levels of lipid droplets, resulting in inhibition of HCV replication [77]. Saito et al. demonstrated that K67, a previously introduced Nrf2 inhibitor, was able to reduce the expression of Nrf2 target genes such as NQO1, GCLC, and UGDH. However, this drug failed to produce any direct effects on HCV replication [78].

### 4.4. Influenza Virus

Kesic et al. showed suppression of Nrf2 gene expression enhanced influenza virus replication; meanwhile, pharmacological induction of Nrf2 via supplementation such as SFN and EGCG suppressed viral replication [163]. This data indicates a causal relationship between the EGCG-induced activation of Nrf2 and the ability to protect against viral replication [163]. In regard to the antiviral mechanism of action, a viral entry seems to be the plausible step, which is negatively targeted by activation of the Nrf2 pathway [163]. In addition, antioxidant supplementation with EGCG significantly increased the mRNA expression levels of the innate immunity-related genes such as IFN-*β*, RIG-I, and MxA [163]. Nrf2 knockout mice displayed elevated oxidative stress and inflammatory gene expression by an influenza virus infection, further emphasizing the importance of the Nrf2 pathway in protection against influenza virus infection [127]. In addition, Nrf2 overexpression stimulated expression of HO-1 and protected alveolar cells against injury induced by the influenza virus and decreased influenza infection in alveolar cells [44]. In line with the protective role of the Nrf2 pathway in the pathogenesis of an influenza virus infection, Nrf2 knockdown downregulated Nrf2 target genes such as HO-1, MX1, and OAS1 and sensitized alveolar cells to oxidative injury induced by an influenza virus [44]. Carbocisteine, also known as S-carboxymethyl cysteine (S-CMC) or (2R)-2- amino-3-carboxymethyl sulfanyl propanoic acid, is a mucoregulatory drug with an anti-inflammatory property [164, 165]. Carbocisteine was able to activate Nrf2 and enhance Nrf2-mediated antioxidant gene expressions such as GCLC, GCLM, and HO-1 in macrophages [166]. Carbocisteine was also able to decrease the expression of virus nucleoprotein, an indicator of viral replication [166]. In the clinical evaluation of the Nrf2 activator for pulmonary diseases, a short-term ingestion of broccoli sprout homogenates, which are enriched in SFN, was found to significantly reduce an influenza virus-induced inflammation as well as virus quantity in smokers [167]. Compounds isolated from *S. baicalensis* were reported to display an anti-influenza activity through Nrf2 activation [168]. Bakuchiol is a naturally occurring phenolic isoprenoid isolated from the seeds of *Psoralea corylifolia* L. [169–171]. Shoji et al. reported that bakuchiol and a series of compounds from licorice induced Nrf2 activation and upregulated NQO1 and glutathione S-transferase (GSTA3) mRNA levels, resulting in reduced expression of influenza A virus H1N1 mRNAs and proteins [172, 173]. Rupestonic acid, which is extracted from *Artemisia rupestris* L., is a sesquiterpene with inhibitory activities against influenza viruses [174, 175]. A rupestonic acid derivative was shown to inhibit influenza replication by upregulating HO-1 expression through promoting Nrf2 nuclear translocation [176, 177]. In particular, p38 MAPK and ERK1/2 pathways seem to be implicated in this upregulation of HO-1 by a rupestonic acid [176, 177]. In addition, HO-1 induction by this compound augmented antiviral IFN response without HO-1 enzymatic activity [176, 177]. Emodin (1,3,8-trihydroxy-6-methylanthraquinone) is a natural anthraquinone compound from several traditional Chinese medicinal plants [178, 179]. Emodin was also found to have inhibitory effects on influenza virus replication through an enhanced Nrf2 signal, resulting in the upregulation of Nrf2 target genes such as HO-1, NQO1, GSH, SOD, GR, CAT, and GPx as well as reduction of virus-induced oxidative stress [180]. Curcumin is a major active compound of turmeric and is commonly used as a coloring agent and spice in foods [181, 182]. Curcumin was shown to inhibit the replication of the influenza virus and virus-induced oxidative stress in vitro [183]. This inhibitory effects of curcumin on the influenza virus seem to be mediated via the enhanced Nrf2 signal and increased IFN-*β* production via the HO-1 pathway after influenza virus infection [183].

### 4.5. RSV

RSV infection is associated with a severe lower respiratory illness characterized by bronchiolitis and respiratory failure. It is the leading cause of infant hospitalization [85]. Loss of Nrf2-dependent antioxidant expression in Nrf2 knockout mice has exacerbated lung inflammation and injury [124–126, 184]. Cho et al. showed the antiviral activity of Nrf2 in a murine model of RSV infection [19]. In this report, RSV-infected Nrf2 knockout mice displayed delayed viral clearance, potentiated viral replication, and enhanced body weight loss with reduced expression of Nrf2 target genes such as GCLC, NQO1, HO-1, GST, and GPx [19]. In addition, bronchoalveolar injury and inflammation were more pronounced in these mice [19]. Overall, exacerbation of lung histopathologic phenotypes was observed in these mice. On the contrary, pretreatment of SFN, an Nrf2 activator, suppressed RSV infection and lung inflammation in normal mice but not in Nrf2 knockout mice [19]. These data suggested a key role for the Nrf2 pathway in host defense against RSV [19]. Mata et al. showed the antiviral activity of roflumilast, a phosphodiesterase 4 inhibitor, against RSV infection [88]. In this study, roflumilast alleviated viral burden following RSV infection, reduced RSV-induced intercellular adhesion molecule- (ICAM-) 1 expression, and restored cilia motility in well-differentiated human bronchial epithelial cells [88]. In addition, roflumilast reversed the reduction of Nrf2, HO-1, and GPx mRNA levels [88]. Roflumilast inhibited RSV infection and mitigated the cytopathological changes associated with RSV infection [88]. Butylated hydroxyanisole (BHA) and its metabolite tert-butyl hydroquinone (tBHQ) have been shown to increase HO-1, NQO1, and Nrf2 protein expressions, with significant amelioration of RSV-induced oxidative stress in both primary and cultured cells [90].

### 4.6. HBV

Protzer et al. showed the antiviral activity of HO-1 in HBV infection [185]. In this paper, HO-1 was either induced by cobalt-protoporphyrin-IX or overexpressed by adenoviral gene transfer [185]. This HO-1 induction protected mice from HBV-induced liver injury and prevented HBV replication [185]. In addition, increased inflammation and liver cell injury in the model of acute hepatitis B were also ameliorated by HO-1 induction [185]. Based on these results, they suggested the induction of HO-1 as a novel therapeutic option for control of inflammation induced by HBV infection [185].

### 4.7. Herpes Virus

Schachtele et al. reported negative modulation of experimental herpes encephalitis-associated neurotoxicity through SFN treatment [105]. In this study, SFN protected mixed neural cultures from HSV-stimulated microglial toxicity through induction of Nrf2 target genes such as HO-1, GPx, GCLM, and GSH [105]. In addition, systemic SFN injections reduced brain inflammation and ROS production in vivo [105].

### 4.8. DENV

DENV infection was shown to activate the Nrf2 pathway in mononuclear phagocytic cells [47]. Treating cells with all-trans retinoic acid (ATRA), a potent inhibitor of Nrf2, significantly decreased the DENV-induced Nrf2 activity [47]. In addition, ATRA inhibited c-type lectin domain family 5, member A (CLEC5A) and tumor necrosis factor- (TNF-) *α* expressions. This led to an increase in the survival rate in suckling mice during DENV infection [47]. HO-1 induction by CoPP, andrographolide, and lucidone all suppressed DENV replication, induced HO-1 expression, and delayed DENV-induced lethality in the suckling mouse model through upregulation of biliverdin, IFN-*α*, OASs, and PKR and downregulation of NS2B/NS3 protease [150, 162]. They also significantly increased the IFN-mediated antiviral response in vitro and in vivo [150, 162].

### 4.9. SVCV

Shao et al. showed pharmacological activation of Nrf2 with SFN and bardoxolone enhanced the cellular total antioxidative capacity by upregulation of SOD1 and HO-1 and dampened SVCV replication [116]. Knocking down the expression of Nrf2 produced the opposite effects [116].

### 4.10. Coxsackievirus

Zhang et al. found that an isatin derivative was able to inhibit coxsackievirus replication through the Nrf2-dependent upregulation of NQO1 and GCLM [186]. Melittin is a major polypeptide in honey bee venom that has been traditionally used against inflammation. Wang et al. showed that melittin was able to ameliorate coxsackievirus-induced myocarditis via activation of the Nrf2 pathway, resulting in an increased expression of HO-1, NQO1, and GCL [187].

### 4.11. Zika Virus

Huang et al. showed that hemin is able to induce HO-1 expression via the Nrf2 pathway and this leads to inhibition of Zika virus replication [188]. This inverse correlation between hemin-induced HO-1 levels and ZIKV replication could be utilized to stimulate an innate cellular response against Zika virus infection.

### 4.12. VSV

Vesicular stomatitis virus (VSV) is a prototypical oncolytic virus that has demonstrated potent oncolytic activity in preclinical models and is being evaluated in clinical trials [189–191]. SFN enhanced VSV replication and oncolysis in PC-3 cells [39]. SFN-VSV combination therapy delayed tumor progression and improved survival in xenograft animal experiments [39]. SFN treatment seemed to dampen the innate antiviral response to assist VSV replication [39].

### 4.13. Theiler Virus

Intracerebral injection of Theiler's murine encephalomyelitis virus (TMEV) into susceptible strains of mice causes a chronic demyelinating disease [192]. Kobayashi et al. showed that DMF suppressed TMEV-induced demyelinating disease by activating the Nrf2 pathway, resulting in upregulation of HO-1, NQO1, and GCLC [193].

### 4.14. RHDV

Rabbit hemorrhagic disease virus (RHDV) is used as an experimental model to study fulminant hepatic failure [194]. Melatonin was shown to activate the Nrf2 pathway with the increased expression of SOD, GPx, and GST. This activation of the Nrf2 pathway by melatonin prevented a fulminant hepatic failure induced by RHDV infection [195].

### 4.15. Porcine Circovirus Type 2

Porcine circovirus type 2 (PCV2), a single-stranded DNA virus, is the primary causative agent of several syndromes collectively known as porcine circovirus disease [196]. Gan et al. showed that overexpression of pig selenoprotein S blocked the ochratoxin-induced promotion of PCV2 replication by inhibiting oxidative stress and p38 phosphorylation in PK15 cells [197].

## 5. Concluding Remarks

In this paper, the roles of virus-induced oxidative stress in the viral life cycle and the pathogenesis of viral diseases were reviewed. Particularly, the cellular management of this virus-induced oxidative stress by cellular utilization of the Nrf2 pathway and its implications in the viral replication and the progression of the viral diseases were explained. In addition, examples of positive and negative regulations of the Nrf2 pathway by a number of pathogenic viruses were described. Finally, various methods of pharmacological and genetic modulations of the Nrf2 pathway as a potential therapeutic option were listed. Considering the significant impact of the Nrf2 pathway on the pathophysiology of both host cell and virus, Nrf2 modulators may be able to serve as a promising supplement for viral diseases by therapeutic modulation of virus-induced oxidative stress in the near future.

## Figures and Tables

**Table 1:** Summary of positive and negative regulations of the Nrf2 pathway by various pathogenic viruses.

Virus	Disease	Host cells or animals	Viral ROS inducer	ROS level	Effect on redox defense system	Effect on Nrf2	Effect on Nrf2 target genes	Effect on others	Reference
MoMuLV ts1	Neurodegenerative disease	Immortalized astrocytes	gPr80env	H_2_O_2_↓	GSH↑, cysteine↑, *γ*-GT↑, CAT↑	↑	xCT↑, GCLC↑, GCLM↑, GPx↑	Sensitivity to cystine deprivation↑, Bcl-2↑, xCT antiporter↑, GRP78↑, GRP94↑, CalR↑	Qiang et al. [58]

HIV	Neurodegenerative disease	Astrocytes	gp120	↑	ND	↑	NQO1↑, HO-1↑	Ca^2+^↑	Reddy et al. [8, 9]
	Neurocognitive disorder	Human neuroblastoma cells	Tat	H_2_O_2_↑	ND	↑	NQO1↑, CAT↑, SOD1↑, SOD2↑, HO-1↑	NMDA receptor↑, SMO↑, spermidine↑	Mastrantonio et al. [65]
	AIDS	HeLa-CD4-LTR-*β*-galactosidase cells	Tat	↑	GSH↓	↑	NQO1↑, HO-1↑, AKR1C1↑	Tat-induced LTR transactivation↓, NF-*κ*B↑	Zhang et al. [63], Zhang et al. [64]
	Respiratory disorder	Alveolar epithelial cells	gp120, Tat	↑	GSH↓	↓	ND	TER↓	Fan et al. [51]
	Respiratory disorder	Monocyte-derived macrophages	gp120, Tat	ND	ND	↓	NQO1↓, GCLC↓	H_2_O_2_ scavenging↓, phagocytosis↓	Staitieh et al. [53]
	AIDS	Transgenic rats	gp120, Tat	↑	ND	↓	HO-1↓	Senescence↑	Davinelli et al. [50]

HCV	Viral hepatitis	Hepatoma cells	ND	ND	ND	Phosphorylation↑	NQO1↑, HO-1↑, GCL↑	p38↑, JNK↑, phospho-Bad↑, phospho-Akt↑	Burdette et al. [48]
	Viral hepatitis	Hepatoma cells	Core, NS5A	↑	ND	Nuclear localization↑	HO-1↑, NQO1↑	PKC↑, CK2↑, PI-3K↑	Ivanov et al., 2011; Smirnova et al., 2016
	Viral hepatitis	Persistently infected hepatoma cells	ND	ND	ND	Phosphorylation↑, nuclear localization↑	NQO1↑, GCLC↑, Maf G↑, G6PD↑, MTHFD2↑, ASNS↑	LD↑, cholesterol↑, triacylglycerol↑	Sugiyama et al. [77]
	HCC	Hepatoma cells	ND	ND	GSH↑	↑	PGD↑, GCLC↑, NQO1↑, UGDH↑	Tolerance to anticancer drug↑, phospho-p62↑, interaction with KEAP1↑	Saito et al. [78]; Medvedev et al., 2017
	Viral hepatitis	Hepatoma cells	Core, NS3	↑	ND	↓	NQO1↓, GCLC↓, GPx↓	sMaf↑, sMaf delocalization↑, proteasomal activity↓, PSMB5↓, sMaf-NS3 interaction↑	Carvajal-Yepes et al. [49]

Influenza virus	Respiratory disease	Human primary alveolar type I-like cells	ND	↑	ND	Nuclear localization↑	HO-1↑	Caspase 1↑, caspase 3↑, IL-8↑	Kosmider et al. [44]
	Respiratory disease	Human bronchial adenocarcinoma cells	ND	↑	ND	Phosphorylation↓	ND	Fibronectin↓	Simon et al. [84]

RSV	Respiratory disease	Human alveolar type II-like epithelial cells and small airway epithelial cells	ND	↑	GSH↓	↓	SOD1↓, SOD2↑, SOD3↓, GST↓, CAT↓, GPx↓	F2-isoprostane↑, NOX↑, RANTES↑, IL-8↑	Hosakote et al., 2009
	Respiratory disease	Nrf2(+/+) mice	ND	↑	ND	Nuclear ARE binding↑↓	GCLC↑↓, UGT1↑↓, NQO1↑↓, HO-1↑↓, GST↑↓, GPx↑↓	ND	Cho et al. [19]
	Respiratory disease	Murine bronchoalveolar lavage, patient samples	ND	↑	GSH↓	Nuclear form↓	SOD1↓, SOD3↓, CAT↓, GST↓, GPx↓	F2-isoprostane↑, MDA↑	Hosakote et al. [20], Komaravelli and Casola [89]
	Respiratory disease	Human bronchial epithelial cells	ND	↑	ND	ND	HO-1↓, GPx↓	ICAM-1↑, b-tubulin↓, Foxj1↓, Dnai2↓, MUC5AC↑, hCLCA1↑, IL-13↑, IL-8↑, IL-6↑, TNF-*α*↑	Mata et al. [88]
	Respiratory disease	Human alveolar type II-like epithelial cells (A549) and small airway epithelial cells	ND	↑	ND	↓	NQO1↓, CAT↓, SOD1↓	CBP↓, HDAC1↑, HDAC2↑	Komaravelli et al. [90]
	Respiratory disease	Human alveolar type II-like epithelial cells and small airway epithelial cells	ND	ND	ND	↓	CAT↓, SOD1↓	Nrf2-SUMO2/3↑, PML↑, Nrf2-PML↑	Komaravelli et al. [43]

HBV	Viral hepatitis	HepG2.2.15, HepAD38	HBx and LHBs	↓	8-OHdG↓, DNPH↓	↑	NQO1↑, GPx↑, GCL↑	c-Raf↑, constitutive proteasome↑, immunoproteasome↓, Mafs↓	Schaedler et al. [99]
	Viral hepatitis	HepG2 cells	HBx	↑	ND	↑	HO-1↑, NQO1↑	p-ATM↑, p-Chk2↑, PKC-d↑	Matsuda et al. [100]
	Viral hepatitis	HepG2, HepG2.2.15, Huh7	HBx	ND	ND	↑	G6PD↑, NQO1↑, GST↑, Cyp2a5↑	p62-KEAP1↑, immobilized KEAP1↑, cell proliferation↑	Liu et al. [46]
	Viral hepatitis	HepG2, Huh7.5, HepAD38, HBV transgenic mice	ND	ND	ND	↑	NQO1↑, GCLC↑, HBsAg↑	PCNA↓, insulin receptor inside↑, insulin receptor surface↓, ALT↑, AST↑, insulin binding↓, *α*-taxillin↑, insulin response↓	Barthel et al. [101]
	Viral hepatitis	Huh7.5 and HepG2	HBsAg	↑	ND	↓	NQO1↓, AP1↓, GPx↓	HBsAg↓, HBeAg↓, subviral particles accumulate at ER due to preS1preS2 domain	Peiffer et al. [52]

HSV	Encephalitis-associated neurotoxicity	Herpes encephalitic mice, microglial cells	ND	↑	GSH↓	↑	HO-1↑, GPx↑	ND	Schachtele et al. [105]

HCMV	Congenital abnormalities	Human foreskin fibroblasts	ND	ND	GSH↑	↑	SOD↑, GPx↑, GCLC↑, HO-1↑, NQO1↑	Maintenance of mTOR activity	Tilton et al. [106]
	Congenital abnormalities	Primary human foreskin fibroblasts	Viral gene products	↑	ND	↑	HO-1↑, GCLC↑	IE1↑, IE2↑, CK2↑, cell viability↑, GSH↑	Lee et al. [11, 42]

KSHV	Sarcoma	Human dermal microvascular endothelial cells	ORF71, vFLIP	↑	ND	Nuclear localization↑	HO-1↑, NQO1↑, GCL↑	LANA-1↑, Src↑, PKC↑, VEGF↑, COX-2↑, PGE2↑, LANA1-Nrf2↑, KEAP1-Nrf2↑	Gjyshi et al. [45]

DENV	Fever	Monocyte-derived dendritic cell	ND	↑	↑	↑	HO-1↑, NQO1↑, SOD2↑, GCLM↑, GCLC↑	IRF3↑, STAT1↑, RIG-I↑, IFIT1↑, LANA-1↑, NOX↑, TNF-*α*, IL-6↑, IFN-*β*1↑, ISG↑, annexin V↑	Olagnier et al. [39]
	Fever	Mononuclear phagocytes	NS2B3	ND	↑	↑	ND	CLEC5A↑, TNF-*α*↑, ER stress↑, PERK↑	Cheng et al. [47]

Marburg virus	Hemorrhagic fever	293T, Huh7, and Vero E6 cells	VP24	ND	ND	Nuclear localization↑	HO-1↑, NQO1↑, GCLM↑	VP24-KEAP1 Kelch domain interaction↑, VP24-KEAP1 mislocalization	Page et al. [113], Edwards et al. [112]
	Hemorrhagic fever	Human monocytic cells	VP24	ND	ND	↑	ND	VP24 dimmerization↓, VP24 monomer cysteines-KEAP1↑	Johnson et al. [114]

SVCV	Spring viremia	Epithelioma papulosum cyprini cells	ND	ND	↑	↑	HO-1↑, SOD1↑	8-OHdG↑, FRAP↑	Yang et al. [117], Shao et al. [116]

WNV	Arboviral encephalitis	BHK cells	ND	↑	GSH↑	↑	GCLC↑, SOD↑, GPx↑	Arsenite-induced stress granule↓, ATF4↑, arsenite-induced mitochondrial damage↓, mitochondrial morphology change↑	Basu et al. [121]

RGNNV	Fish disease	The grouper fin cells	ND	↑	SOD↑, CAT↑	↑	SOD↑, CAT↑	Apoptosis↑, mitochondrial potential↓	Chang et al. [123]

Virus names, diseases caused by viruses, host cells or animals used for infection, ROS level after infection, effect on the redox defense system, effect on Nrf2 and its target genes, and other effects were listed accordingly. ND indicates “not determined.” Abbreviations used within the table are as follows. 8-OHdG: 8-hydroxydeoxyguanosine; AIDS: acquired immunodeficiency syndrome; AKR1C: aldo-ketoreductase 1C1; Akt: protein kinase B; ALT: alanine aminotransferase; AP-1: transcription factor composed of cFos and cJun dimer; ARE: antioxidant response element; ASNS: asparagine synthetase; AST: aspartate aminotransferase; ATM: ATM serine/threonine inase; Bad: bcl-2-associated death promoter; Bcl-2: B-cell lymphoma 2; CalR: calreticulin; CAT: catalase; CBP: CREB-binding protein; Chk2: checkpoint kinase 2; CK2: casein kinase 2; CLEC5A: c-type lectin domain family 5, member A; COX-2: cyclooxygenase 2; c-Raf: RAF protooncogene serine/threonine-protein kinase; Cyp2a5: cytochrome P450 2A5; DENV: dengue virus; Dnai2: dynein intermediate chain 2; DNPH: 2,4-dinitrophenylhydrazine; Foxj1: forkhead box protein J1; FRAP: ferric reducing ability of plasma reagent; G6PD: glucose 6 phosphate dehydrogenase; GCLC: glutamate cysteine ligase catalytic subunit; GCLM: glutamate cysteine ligase regulatory subunit; *γ*-GT: gamma-glutamyl transpeptidase; GPx: glutathione peroxidase; GRP78: glucose-responsive proteins 78; GRP94: glucose-responsive proteins 94; GSH: glutathione; GST: glutathione S transferase; HBeAg: hepatitis B e antigen; HBsAg: hepatitis B surface antigen; HBx: hepatitis B virus protein X; HCC: hepatocellular carcinoma; hCLCA1: human chloride channel accessory 1; HCMV: human cytomegalovirus; HCV: hepatitis C virus; HDAC: histone deacetylase; HIV: human immunodeficiency virus; HO-1: heme oxygenase; HSV: herpes simplex virus; ICAM-1: intercellular adhesion molecule 1; IE1: immediate early protein 1; IE2: immediate early protein 2; IFIT1: interferon-induced protein with tetra-tricopeptide repeats 1; IFN: interferon; IL-13: interleukin 13; IL-6: interleukin 6; IL-8: interleukin 8; IRF: IFN regulatory factor 3; ISG: interferon-stimulated gene; JNK: c-Jun kinase; KEAP1: Kelch-like ECH-associated protein 1; KSHV: Kaposi's sarcoma-associated herpesvirus; LANA-1: latency-associated nuclear antigen 1; LD: lipid droplet; LHBs: hepatitis B virus large surface protein; LTR: long terminal repeat; Maf: transcription factor Maf; MDA: malondialdehyde; MoMuLV ts1: Moloney murine leukemia virus ts1; MTHFD2: methylenetetrahydrofolate dehydrogenase 2; mTOR: mammalian target of rapamycin; MUC5AC: mucin 5AC; NF-*κ*B: nuclear factor kappa-light-chain-enhancer of activated B cells; NMDA: N-methyl-D-aspartate; NOX: NADPH oxidase; NQO1: NAD(P)H dehydrogenase quinone 1; NS2B3: nonstructural protein 2B3; NS3: nonstructural protein 3; NS5A: nonstructural protein 5A; ORF71: open reading frame 71; PCNA: proliferating cell nuclear antigen; PERK: protein kinase R-like ER kinase; PGD: phosphogluconate dehydrogenase; PGE2: prostaglandin E2; PI3K: phosphatidyl inositol 3 kinase; PKC: protein kinase C; PML: promyelocytic leukemia protein; PSMB5: proteasome subunit beta type-5; RANTES: regulated on activation, normal T cell expressed and secreted; RGNNV: red-spotted grouper nervous necrosis virus; RIG-I: RIG-I-like receptor; RSV: respiratory syncytial virus; sMaf: small musculoaponeurotic fibrosarcoma; SMO: spermine oxidase; SOD: superoxide dismutase; Src: protooncogene tyrosine-protein kinase; STAT1: signal transducer and activator of transcription 1; SUMO: small ubiquitin-like modifier; SVCV: spring viremia of carp virus; TER: transepithelial electrical resistance; TNF-*α*: tumor necrosis factor alpha; UGDH: UDP-glucose dehydrogenase; VEGF: vascular endothelial growth factor; vFLIP: viral FLICE-like inhibitory protein; WNV: West Nile virus; xCT: cell surface cysteine-glutamate antiporter.

**Table 2:** Summary of pharmacologic and genetic Nrf2 modulators and their effects on Nrf2, cellular ROS level, viral pathogenesis, virus replication, and other parameters.

Virus	Pharmacologic or genetic modulation	Target cells or animals	Effect on Nrf2	Effect on Nrf2 target genes	ROS level	Effect on pathogenesis	Effect on virus	Effect on others	Reference
MoMuLV ts1	*α*-Luminol	Astrocytes, FVB/N mice	↑	GPx↓, xCT↓	H_2_O_2_↓	↓	Replication↓, gPr80env↓	Cellular DNA synthesis↑, GSH↑	Jiang et al. [40]
		Thymocyte, FVB/N mice	↑	ND	↓	↓	gPr80env↓	CK8/5 gradient↑, caspase-3↓, cell number↑	Scofield et al. [129, 130]
		Intestinal lymphoid cells, FVB/N mice	↑	ND	ND	↓	gPr80env↓, gp70↓	CD4 T cell number↑, CD8 T cell number↑, caspase-3↓, TSH gradient↑, Ki67 gradient↑	Scofield et al. [129, 130]
		Astrocytes, neurons, FVB/N mice	↑	ND	ND	↓	ND	ND	Reddy et al., 2010
	Minocycline	Astrocytes, neurons, thymocytes	↑	ND	↓	↓	↓ in CNS	GFAP↓, astrocyte cell number↑, MDA↓, Ik-B*α*↑, p65↓, COX-2↓, CD68↓, caspase-3↓, p53↓	Kuang et al. [131]

HIV	EGCG	HeLa-CD4-LTR-*β*-galactosidase indicator cells	↑	↑	↓	ND	ND	Tat-induced LTR transactivation↓, GSH↑, NF-*κ*B↓, NOX2↓, AKT↓, AMPK↑	Zhang et al. [64]
	Tanshinone II A	HeLa cells	↑	↑	↓	ND	ND	GSH↑, Nampt↑, NAD+↑, AMPK↑, SIRT1↑, Tat-induced LTR transactivation↓	Zhang et al. [62, 186]
	Nrf2 silencing, BSO	Alveolar epithelial cells, HIV transgenic rats	↓	GSSG↓, GCLC↓, GST↓, GSR↓, NQO1↓	ND	↓	ND	Paracellular permeability↑, TER↓, ZO-1↓, occludin↓, claudin-18↓	Fan et al. [51]
	Nrf2 overexpression, SFN, GSH	Alveolar epithelial cells, HIV transgenic rats	↑	NQO1↑, GSH↑	ND	↓	ND	Paracellular permeability↓, TER↑, ZO-1↑, occludin↑	Fan et al. [51]
	DMF	Monocyte-derived macrophages	↑	HO-1↑, GPx↑, NQO1↑	ND	↓	↓	NF-*κ*B↓, TNF-*α*↓	Cross et al. [146]
	Sulforaphane, EGCG, DMF	Primary macrophage	↑	ND	ND	ND	↓ in macrophage after entry	ND	Furuya et al. [145]
	Sulforaphane	Monocyte-derived macrophage	↑	NQO1↑, GCLC↑	ND	ND	ND	Phagocytic function↑	Staitieh et al. [53]

HCV	Biliverdin	Hepatoma cells	ND	ND	ND	ND	NS3/4A protease↓, replication↓	ND	Zhu et al. [148]
	HO-1 induction by CoPP, HO-1 overexpression, CO, Fe	Hepatoma cells	ND	HO-1↑	ND	ND	↓	OAS↑, PKR↑, IFN-*α*↑, HRI↑	Lehmann et al. [149]
	Lucidone	Hepatoma cells	↑	HO-1↑	ND	ND	Replication↓	OAS↑, PKR↑, IFN-*α*↑, bilirubin↑	Chen et al., 2013
	Andrographolide	Hepatoma cells	↑	HO-1↑	ND	ND	NS3/4A protease↓, replication↓	p38↑, bilirubin↑, OAS↑, PKR↑, IFN-*α*↑, HRI↑	Lee et al. [153]
	Fluvastatin	Hepatoma cells		HO-1↑			Replication↓	Bach1↓, KLF2↑, matrix stiffness↑	Wuestenberg et al. [157]
	SFN	Hepatoma cells	↑	HO-1↑	ND	ND	NS3/4A protease↓, replication↓	PI3K↑, PKR↑, OAS↑, IFN-*α*↑, bilirubin↑	Yu et al. [158]
	Celastrol	Hepatoma cells	↑	HO-1↑	ND	ND	NS3/4A protease↓, replication↓	JNK↑, IFN-*α*↑, OAS↑	Tseng et al. [162]
	Nrf2 silencing	Hepatoma cells	↓	NQO1↓, GCLC↓, G6PD↓	ND	ND	Replication↓	LD↓, cholesterol↓, triacylglycerol↓, phospholipid↓	Sugiyama et al. [77]
	K67	Hepatoma cells	↓	NQO1↓, GCLC↓, UGDH↓		↓	ND	KEAP1-phospho-p62 interaction↓, tumor growth↓, tolerance to anticancer agents↓	Saito et al. [78]

Influenza virus	Nrf2 silencing	Nasal epithelial cell	↓	HO-1↓	ND	ND	Replication↑, titer↑	ND	Kesic et al. [163]
	EGCG and SFN	Nasal epithelial cell	↑	HO-1↑	↓	↓	Replication↓, entry↓	IFN-*α*↑, RIG-I↑, MxA↑	Kesic et al. [163]
	Nrf2 deficiency and cigarette smoke	Nrf2-deficient mice	↓	NQO1↓, GCLC↓, HO-1↓	↑	↑	ND	TNF-*α* ↑, KC↑, IFN-*α*, NF-*κ*B↑, lung permeability damage↑, mucus production↑, macrophages↑, neutrophils↑, cell injury↑	Yageta et al. [127]
	Nrf2 expression	Human primary alveolar type I-like cells	↑	HO-1↑	↓	↓	Replication↓	GSH↑, IL-8↑, cell injury↓	Kosmider et al. [44]
	Nrf2 deficiency	Human primary alveolar type I-like cells	↓	HO-1↓, MX1↓, OAS1↓	↓	↓	Replication↓	Caspases 1 and 3↑, cell injury↓	Kosmider et al. [44]
	Carbocisteine	Nrf2-deficient mice, macrophages	↑	GCLC↑, GCLM↑, HO-1↑	↓	↓	Replication↓	PI3K↑, 8-OHdG↓, mucus production↓	Yageta et al. [166]
	Broccoli sprouts (SFN)	Human subjects (smoker, nonsmoker)	↑	ND	ND	↓	Replication↓	Inflammation↓, IL-6↓	Noah et al. [167]
	Compounds from S. baicalensis	Nrf2/ARE luciferase reporter (HepG2C8), MDCK	↑	ND	ND	ND	Replication↓	ND	Ji et al. [168]
	Bakuchiol	MDCK	↑	NQO1↑, GST↑	ND	ND	Infection↓, viral titer↓	IFN-*α*↓, Mx1↓	Shoji et al. [172]
	Compounds from licorice (echinatin)	HepG2	↑	HO-1↑, NQO1↑	ND	ND	Replication↓	ALT↓, AST↓, GSH↓	Ji et al. [173]
	Rupestonic acid deriv, YZH-106 from CYZH pellet	MDCK, RAW264.7	Nuclear translocation↑	HO-1↑	↓	↓	Replication↓	p38↑, ERK1/2↑, IFN-*α*↑, IFN-*β*↑, IFIT1↑, IFITM3↑, OAS1↑, PKR↑	Ma et al. [176], Yin et al. [177]
	Emodin	A549 cells	↑	HO-1↑, NQO1↑, GSH↑, SOD↑, GR↑, CAT↑, GPx↑	↓	↓	Replication↓	TLR2/3/4/7↓, MyD88↓, TRAF6↓, phospho-p38↓, phospho-JNK↓, p65↓, IL-1*β*↓, TNF-*α*↓, IL-6↓	Dai et al. [180]
	Curcumin	A549 cells	↑	HO-1↑, GSH↑, NQO1↑, GSTA3↑	↓	↓	Replication↓, viral titer↓	IFN-*β*↑, TLR2/3/4/7↓, MyD88↓, TRAF6↓, phospho-Akt↓, phospho-p38↓, phospho-JNK↓, TNF-*α*↓, IL-1*β*↓, IL-6↓, IL-8↓, NF-*κ*B↓, MMP-2↓, MMP-9↓	Dai et al. [183]

RSV	Nrf2 deficiency	Bronchoalveolar lavage fluid of Nrf2-deficient mice	↓	GCLC↓, UGT1↓, NQO1↓, HO-1↓, GST↓, GPx↓	ND	↑	Replication↑, viral titer↑	Nasal airway injury↑, mucus production↑, IL-6↑, IL-13↑, GSH↓, GSSG↓, protein oxidation↑, AP-1↑, NF-*κ*B↑, DNP↑	Cho et al. [19]
	SFN	Bronchoalveolar lavage fluid	↑	NQO1↑, GST↑, HO-1↑, GPx↑	ND	ND	Replication↓	Neutrophils↓, eosinophils↓	Cho et al. [19]
	Roflumilast N-oxide	Bronchial epithelia cell	↑	HO-1↑, GPx↑	↓	↓	Replication↓	ICAM-1↓, *β*-tubulin↑, Foxj1↑, Dnai2↑, MUC5AC↓, hCLCA1↓, IL-13↓, IL-8↓, IL-6↓, TNF-*α*↓	Mata et al. [88]
	Butylated hydroxyanisole	Bronchial epithelia cell	↑	HO-1↑, NQO1↑, CAT↑, SOD1↑	↓	↓	ND	8-Isoprostaine↓	Komaravelli et al. [90]

HBV	HO-1 induction (CoPP)	Mouse infection model, HepG2 cells		HO-1↑	ND	↓	Core↓, HBeAg↓, HBV DNA↓	ALT↓	Protzer et al. [185]

Herpes virus	Sulforaphane	Astrocyte, mixed neural culture	↑	HO-1↑, GPx↑, GCLM↑, GSH↑	↓	↓	↔	Macrophage infiltration↓	Schachtele et al. [105]

DENV	ATRA	Mononuclear phagocytes	↓	ND	ND	↓	CLEC5A↓, TNF-*α*↓	ND	Cheng et al. [47]
	HO-1 induction CoPP, andrographolide	Huh-7 cells	ND	HO-1↑	ND	↓	↓	Biliverdin↑, NS2B/NS3 protease↓, IFN-*α*↑, OASs↑, PKR↑	Tseng et al., 2016
	Nrf2 and HO-1 induction (lucidone)	Huh-7 cells	↑	HO-1↑	ND	↓	Replication↓	Biliverdin↑, NS2B/NS3 protease↓, IFN-*α*↑, OASs↑, PKR↑	Chen et al. [150]

SVCV	SFN bardoxolone		↑	SOD↑, HO-1↑	ND	ND	↓	Total antioxidant capacity↑	Yang et al. [117], Shao et al. [116]

Coxsackievirus	Isatin derivative 45	HeLa and HL-1 cells	Nuclear localization↑	NQO1↑, GCLM↑	ND	ND	↓	Procaspase 3 cleavage↓, GRP78↑, UPR↑, p-PERK↑, eIF4GI↑, cap-dependent translation↑	Zhang et al. [62, 186]

Coxsackievirus	Melittin	HeLa cells	↑	HO-1↑, NQO1↑, GCL↑	ND	↓	↓	Apoptosis↓, Bax↓, caspase-3↓, bcl-2↑, HDAC2↑, GSK-3*β*↑, AST, CK, HBDH and LDH↑, IL-1*β*↓, IL-6↓, TNF-*α*↓, MCP-1↓	Wang et al. [187]

ZIKA virus	HO-1 induction (hemin)	Human monocyte-derived macrophage	ND	HO-1↑	ND		↓	ND	Huang et al. [188]

VSV	SFN	PC-3	↑	HO-1↑, NQO1, GCLC↑, GCLM↑, SQSTM1↑		↓	↑	Oncolytic activity↑, tumor progression↓, LC3↑, p62↑, autophagy↑, innate antiviral response↓, IRF3↓, STAT1↓, STING↓	Olagnier et al., 2017

Theiler virus	DMF	TMEV-infected mice	↑	HO-1↑, NQO1↑, GCLC↑	ND	↓	↑	IFN-*γ*, IL-17a, IL-4, IL-10 and TNF-*α*, T cell response↓	Kobayashi et al. [193]
RHDV	Melatonin	New Zealand white rabbits	↑	SOD↑, GPx↑, GST↑	ND	↓	ND	ND	Crespo et al. [195]
PCV2	Selenium, selenoprotein S	PK15	↑	GCL↑, GCSL↑, GSH↑	↓	ND	Ochratoxin A-induced replication↓	Ochratoxin A-induced ROS↓, ochratoxin A-induced p38 phosphorylation↓	Gan et al. [197]

ND indicates “not determined.” Abbreviations used within the table are as follows. Akt: protein kinase B; ALT: alanine aminotransferase; AMPK: AMP-activated protein kinase; AST: aspartate aminotransferase; ATRA: all-trans retinoic acid; BACH1: basic leucine zipper transcription factor; Bax: bcl-2-like protein 4; Bcl-2: B-cell lymphoma 2; BSO: buthionine sulfoximine; CK8/5: casein kinase 8 and 5; CNS: central nerve system; CoPP: cobalt-protoporphyrin-IX; DMF: dimethyl fumarate; Dnai2: dynein intermediate chain 2; DNP: dinitrophenyl; EGCG: epigallocatechin-3-gallate; eIF4GI: eukaryotic translation initiation factor; Foxj1: forkhead box protein J1; G6PD: glucose 6 phosphate dehydrogenase; GCLC: glutamate cysteine ligase catalytic subunit; GCLM: glutamate cysteine ligase regulatory subunit; GFAP: glial fibrillary acid protein; GPx: glutathione peroxidase; GR: glutathione reductase; GRP78: glucose-responsive proteins 78; GSH: glutathione; GSK: glycogen synthase kinase; GSR: glutathione reductase; GSSG: intracellular reduced glutathione-to-glutathione disulfide; GST: glutathione S transferase; HBDH: *β*-hydroxybutyrate dehydrogenase; hCLCA1: human chloride channel accessory 1; HDAC: histone deacetylase; HO-1: heme oxygenase; HRI: heme-regulated eIF2alpha kinase; ICAM-1: intercellular adhesion molecule 1; IFIT1: IFN-induced protein with tetratricopeptider repeats 1; IFITM3: IFN-inducible transmembrane protein 3; IFN: IFN; Ik-B*α*: nuclear factor of kappa light polypeptide gene enhancer in B-cells inhibitor, alpha; IL-13: interleukin 13; IL-6: interleukin 6; IL-8: interleukin 8; IRF3: IFN regulatory factor 3; JNK: c-Jun kinase; KC: keratinocyte-derived chemokine; Ki67: antigen KI-67; KLF2: Krueppel-like factor 2; LDH: lactate dehydrogenase; MCP-1: monocyte chemoattractant protein-1; MDA: malondialdehyde; MUC5AC: mucin 5AC; MX1: IFN-induced GTP-binding protein; MxA: MxGTPases; NAD+: nicotinamide adenine dinucleotide; Nampt: nicotinamide phosphoribosyltransferase; NF-*κ*B: nuclear factor kappa-light-chain-enhancer of activated B cells; NOX: NADPH oxidase; NQO1: NAD(P)H dehydrogenase quinone 1; NS3/4A: nonstructural protein 3/4A; OAS: oligoadenylate synthetase; PCV2: porcine circovirus type 2; PERK: protein kinase R-like ER kinase; PI3K: phosphatidyl inositol 3 kinase; PKR: protein kinase R; RHDV: rabbit hemorrhagic disease virus; SFN: sulforaphane; SIRT1: NAD+-dependent histone deacetylase sirtuin1; SQSTM1: sequestosome-1; STAT1: signal transducer and activator of transcription 1; STING: stimulator of IFN genes; SVCV: spring viremia of carp virus; tBHQ: tert-butyl hydroquinone; TER: transepithelial electrical resistance; TMEV: Theiler's murine encephalomyelitis virus; TNF-*α*: tumor necrosis factor alpha; TSH: thyroid stimulating hormone; UGDH: UDP-glucose dehydrogenase; UPR: unfold protein response; VSV: vascular stomatitis virus; xCT: cell surface cysteine-glutamate antiporter; ZO-1: zonula occludens-1.
